# A Cadaveric Comparative Study on the Surgical Accuracy of Freehand, Computer Navigation, and Patient-Specific Instruments in Joint-Preserving Bone Tumor Resections

**DOI:** 10.1155/2018/4065846

**Published:** 2018-11-13

**Authors:** Sarah E. Bosma, Kwok Chuen Wong, Laurent Paul, Jasper G. Gerbers, Paul C. Jutte

**Affiliations:** ^1^Department of Orthopedics, University Medical Center Groningen, P.O. Box 30.001, 9700 RB Groningen, Netherlands; ^2^Department of Orthopedics, Prince of Wales Hospital, Sha Tin, Hong Kong; ^3^3D-Side, 1348 Louvain-la-Neuve, Belgium

## Abstract

Orthopedic oncologic surgery requires preservation of a functioning limb at the essence of achieving safe margins. With most bone sarcomas arising from the metaphyseal region, in close proximity to joints, joint-salvage surgery can be challenging. Intraoperative guidance techniques like computer-assisted surgery (CAS) and patient-specific instrumentation (PSI) could assist in achieving higher surgical accuracy. This study investigates the surgical accuracy of freehand, CAS- and PSI-assisted joint-preserving tumor resections and tests whether integration of CAS with PSI (CAS + PSI) can further improve accuracy. CT scans of 16 simulated tumors around the knee in four human cadavers were performed and imported into engineering software (MIMICS) for 3D planning of multiplanar joint-preserving resections. The planned resections were transferred to the navigation system and/or used for PSI design. Location accuracy (LA), entry and exit points of all 56 planes, and resection time were measured by postprocedural CT. Both CAS + PSI- and PSI-assisted techniques could reproduce planned resections with a mean LA of less than 2 mm. There was no statistical difference in LA between CAS + PSI and PSI resections (*p*=0.92), but both CAS + PSI and PSI showed a significantly higher LA compared to CAS (*p*=0.042 and *p*=0.034, respectively). PSI-assisted resections were faster compared to CAS + PSI (*p* < 0.001) and CAS (*p* < 0.001). Adding CAS to PSI did improve the exit points, however not significantly. In conclusion, PSI showed the best overall surgical accuracy and is fastest and easy to use. CAS could be used as an intraoperative quality control tool for PSI, and integration of CAS with PSI is possible but did not improve surgical accuracy. Both CAS and PSI seem complementary in improving surgical accuracy and are not mutually exclusive. Image-based techniques like CAS and PSI are superior over freehand resection. Surgeons should choose the technique most suitable based on the patient and tumor specifics.

## 1. Introduction

Orthopedic oncologic surgery requires the preservation of a functioning limb at the essence of achieving safe margins. Up until the 1970s, amputation was the first choice of treatment for bone tumors, with a survival rate of only 11% [1]. Nowadays, with the developments in diagnostic imaging, surgical techniques, and adjuvant therapies (e.g., chemotherapy), the emphasis is on limb-salvage surgery [2, 3]. Limb-salvage surgery aims to preserve as much unaffected tissue as possible without compromising safe tumor margins. This improves local control, benefits the reconstruction, and reduces the morbidity, thereby improving the short- and long-term functional outcomes. With most bone sarcomas arising from the metaphyseal region, in close proximity to joints and neurovascular and visceral structures, joint-salvage surgery could be challenging [4–6]. Surgical accuracy is therefore essential in joint-salvage orthopedic oncologic surgery.

A resection can be performed “freehand.” Using this technique, the surgeon must intraoperatively rely on two-dimensional (2D) preoperatively acquired images (computer tomography (CT) or magnetic resonance imaging (MRI)) and mentally integrate these into a three-dimensional (3D) intraoperative surgical situation [4]. This limited intraoperative guidance might result in surgical inaccuracy in bone resections, as was proven even for experienced surgeons [7, 8]. Computer-assisted surgery (CAS) and patient-specific instrumentation (PSI) have been introduced to address the inaccuracy of tumor resections and improve predictability of bone tumor resections [9].

CAS, or surgical navigation, was initially used to improve pedicle screw insertion in the spine and reduce outliners in component alignment in total knee arthroplasties [10, 11]. Later, it gained more acceptance and showed its advantages in orthopedic oncologic surgery [12]. It allows linking between patients' imaging information and anatomy by tracking a registration of the preoperative images (CT and MRI) and the patient on the operating table. The surgeon can preoperatively plan the bone tumor resection in a 3D virtual scenario and analyze different alternatives for resection. Additionally, during the surgery, there is real-time 3D radiation-free visual feedback [13, 14]. Previous research has shown that CAS aids in achieving adequate margins [15, 16] and improves the accuracy of the osteotomies [4, 6, 17, 18]. However, not all surgical tools are real-time navigated, and a reliable navigated saw is often not available; therefore, this accuracy and precision may be lost when the actual cut is performed, since the human hand is guiding the saw.

PSI was introduced for resection of bony parts during prosthesis placement and osteotomies. Later, it was also introduced to guide tumor resections [19–21]. In the same fashion as for CAS, control over safe margins is provided by accurate preplanning and subsequent accurate intraoperative guiding of the resection planes. One face of the guide is the (negative) surface of the bone representing an automatic matching of the patient with his preoperative images, fitting onto the bone surface in one possible configuration. During the actual cut, the saw blade is aligned with the guide and thereby provides actual guidance during the resection. A possible limitation of PSI is an inaccurate fit to the bone, due to lack of surface characteristics, design flaws, and soft tissue extension that could lead to PSI malposition, all resulting in loss of accuracy. Another limitation is that PSIs rely on sufficient bone exposure and can require a larger dissection.

Thus, CAS has the advantage of providing real-time data on the spatial position but currently not a plane cut due to lack of a reliable navigated saw. Therefore, the accuracy is lost as soon as the human hand comes into play for the actual resection [4, 12–14]. PSI is very accurate in guiding a cutting plane but lacks feedback on its actual position other than the close fit to the bone surface which needs to be cleaned from soft tissue, and PSI needs to be devoid of design flaws. Joint-preserving surgery has been performed in select patients with bone sarcomas of extremities and allows patients to retain the native joint with better joint function [1]. However, it is technically demanding as it is difficult to translate preoperative CT/MR tumor extent to the patients' intraoperative anatomy.

Given the importance of accurate surgical margins in resection of a primary bone sarcoma, a comparative study of different techniques in a cadaveric experimental setting is essential to compare the accuracy of the various techniques. Also to our knowledge, the use of PSI for bone tumor resection in the knee region was not adequately assessed except in the small clinical series of Bellanova et al. [22]. The aim of this study is [1] to evaluate surgical accuracy and bone cutting time of freehand joint-preserving resections compared to navigated and PSI-assisted joint-preserving resections in yet not investigated but most common sites in bone tumor, namely, distal femur and proximal tibia; [2] to verify accurate positioning of PSI using CAS as an intraoperative quality control tool; and [3] to investigate whether integration of CAS and PSI improves surgical accuracy.

## 2. Materials and Methods

**Figure 1:**
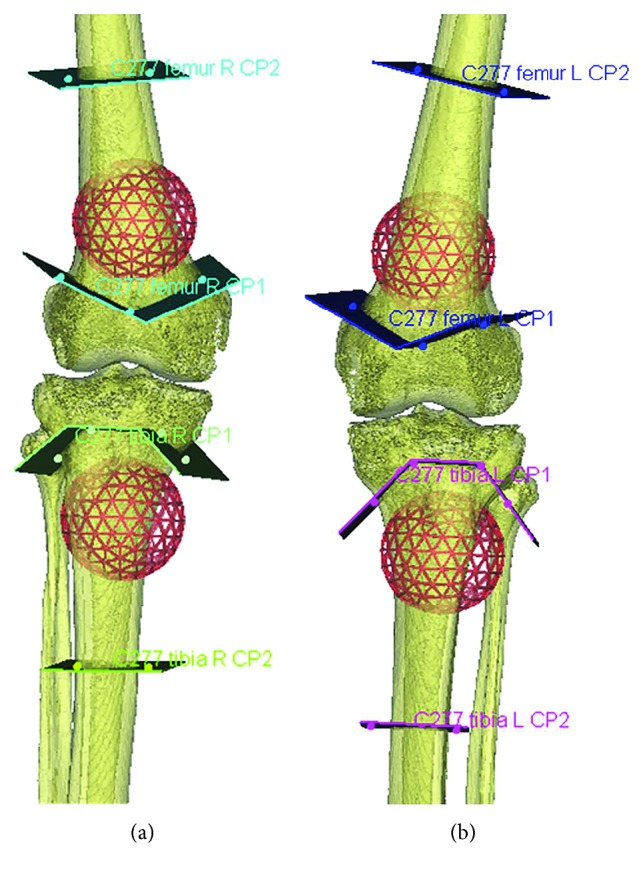
Resection planning as performed in the Mimics engineering software.

Eight distal femurs and eight proximal tibia bone tumors were simulated on four fresh-frozen human cadavers. 56 cutting planes were planned to simulate a joint-preserving tumor resection around the knee. These planes were available for postoperative measurements. Four surgical techniques were performed and compared: freehand, CAS, PSI, and CAS + PSI. Each technique was used twice in the femur and twice in the tibia of the same cadaver. High resolution and sharpness CT scans (Siemens AG Somatom Flash, Forchheim, Germany; software Syngo CT VA48A, Extra Routine ZHR protocol; collimation 0.5 reconstructed to 0.4 mm slice thickness) were acquired from the femur mid-diaphysis to the tibia mid-diaphysis of each leg. The CT images were used for preoperative planning of the resections by using the biomedical engineering software (Mimics 16.0; Materialise, Leuven, Belgium). A computer-aided design (CAD) file of a 40 mm sphere representing a virtual tumor was placed at the metaphysis of the distal femur and proximal tibia. Resection planes (thickness of 1 mm) were consistently positioned onto the virtual 3D tumor bone models across all groups (Figure 1).

The virtual tumor resections were planned to spare the knee joint using a multiplanar cut like in clinical practice. Resection planes close to the joint were determined with a 10 mm tumor margin. Resection planes in the diaphysis were positioned at a 25 mm safe margin. The resection planning was exported as CAD files in the Standardized Tessellation Language (STL) format.

Planning of the resection planes in both knees of one of the cadavers was performed in the Mimics engineering software. A virtual tumor was placed in the metaphysis of the proximal tibia and the distal femur of each knee. Resection planes were multiplanar near the joints with preservation of the ligament attachments.

### 2.1. Patient-Specific Instrumentation

**Figure 2:**
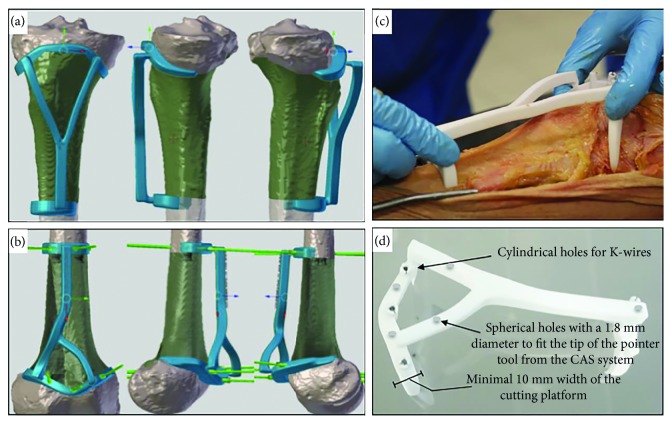
PSI design and its unique features. (a) PSI design of the tibia. (b) PSI design of the femur. The green wires simulate the K-wires and show where and how the PSI will be pinned onto the bone surface. Pin tract design is mostly perpendicular to the bone to avoid shear forces during placement. (c) Each PSI was designed to fit onto a unique position on the bone surface. When the cartilaginous surface was in the cutting trajectory, the PSI was bridging over it, with a minimum of 2 mm, to avoid any contact yielding to a potential malpositioning (cartilage is not visible in a CT acquisition). (d) The width of the cutting platform was around 10 mm, to get a sufficient support for the saw blade. Spherical holes that fit the pointer tool from the CAS system were used to check the position of the guide.

Each PSI was designed to fit in a unique position on the bone surface and endowed a flat surface to indicate the target cutting plane (Figure 2). Every PSI contained cylindrical holes, at the interface of the planes, designed for 2 mm Kirschner wires (K-wires) used to pin the PSI onto the bone. The PSI for the PSI + CAS group was designed with five extra spherical holes (“checkpoints”) with a 1.8 mm diameter to fit the tip of the pointer tool from the CAS system. In the CAS + PSI group, the CAS system used the checkpoints for intraoperative control of the positioning of the PSI guide in relation to the preoperative planning but not the localization of the sites of bone resections. Figures 2–2 show the PSI design and its unique features. After approving the design of the PSI, the PSI was manufactured (3D-Side, Belgium) by additive manufacturing with a selective laser sintering technology (EOS, Krailing, Germany) in an ISO-certified biocompatible polyamide material.

### 2.2. Tumor Resections

**Figure 3:**
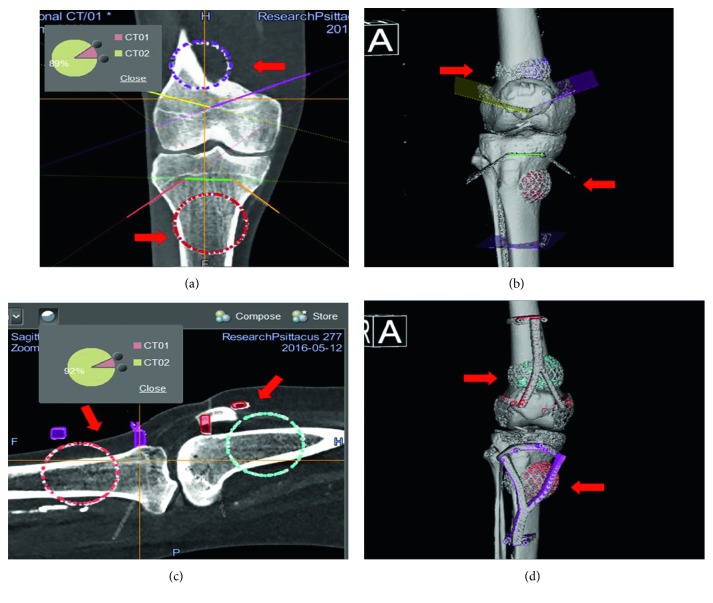
Fusion of the datasets in the Stryker Navigation system. Preoperative images of surgical navigation planning on the navigation display for the CAS group (a, b) and CAS + PSI group (c, d). The original CT image datasets (CT01) were fused with a modified CT dataset (CT02) containing the locations of the tumor, resection planes, and PSI (red arrows) after simulated tumor resections in the MIMICS software. The planned resection planes could be marked with tools in the navigation system. Also, the CAD models (red arrows) of a tumor and PSI could be imported into the navigation system and positioned as planned in the MIMICS software. Reformatted coronal (a) and sagittal (c) images and the reconstructed 3D planning bone models (c, d) with a tumor, resection planes, and PSI illustrate the transfer of the virtual planning in MIMICS to the navigation system by the method of CAD to DICOM conversion and image fusion.

The Stryker Navigation system (OrthoMap 3D®) was used for the navigation-assisted resections. The virtual resection planes in the Mimics software were transferred to the navigation system by a method described by one of the authors in a previous study [23, 24]. Planning, performed in Mimics, was exported to modified Digital Imaging and Communications in Medicine (DICOM) images that contain the original CT images, tumor extent, PSI design, and resection planes. The original DICOM dataset of the cadavers and the modified DICOM dataset were imported into OrthoMap 3D®. After both datasets were fused together, the planned resection planes could then be marked with tools and the CAD model of the PSI could be positioned in the navigation system as planned in the MIMICS software.  Figure 3 provides screenshots from the Stryker Navigation system of the previously mentioned steps.

One cadaver has been used for each resection method, and four resections were performed on each cadaver. Resections were performed by two senior orthopedic tumor surgeons who have experience with both CAS and PSI (KCW and PCJ). Each surgeon performed two resections for each of the four different techniques, one in the femur and one in the tibia of each cadaver.

Surgical exposure was performed according to the standard clinical procedure, with an extended medial approach to expose both femur and tibia.

### 2.3. Freehand Resection

**Figure 4:**
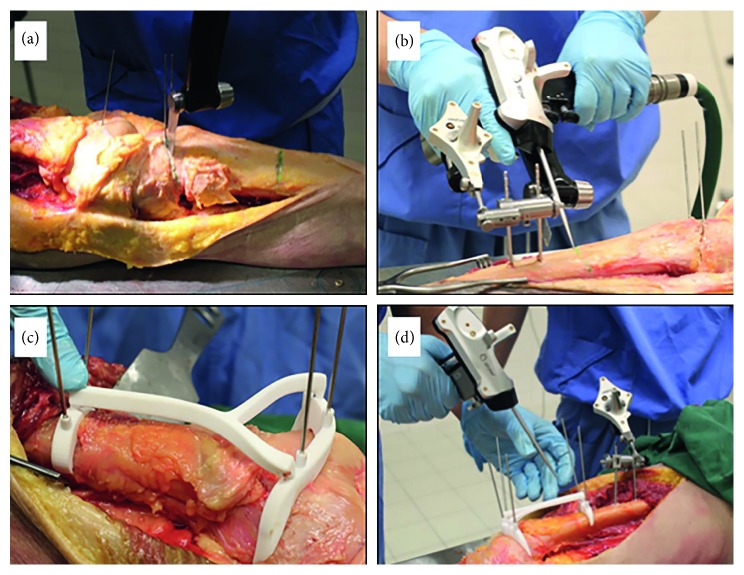
The techniques used in this experiment. (a) The freehand procedure. (b) The CAS-assisted technique. A pointer (in the surgeons right hand) is used to determine what the direction of the plane is and how to align the saw (in the surgeons left hand). (c) How the PSI is pinned onto the bone is shown. (d) How the position of the guide is checked by using CAS is shown.

Entry lines of the cutting planes were based on measurements performed on the Mimics software (Figure 4). K-wires were placed at the intersections of the planes, and the resection was executed manually with an oscillating saw without guidance.

### 2.4. Navigation-Assisted Resection

CAS registration was performed according to the standard clinical workflow (Figure 4). A patient tracker was placed on the bone (proximal femur or distal tibia), and navigation instruments were calibrated. Image-to-patient registration (correlation of spatial coordinates between patients' anatomy and preoperative CT images) has been performed by surface matching. The system generated a registration error after this manual registration. Real-time matching between the bone anatomy and the virtual images was then assessed by running the navigation pointer on the bone surface or by checking specific known and well-defined anatomic landmarks. The navigation system was considered accurate only if there was exact matching between the image on the navigation console and the patient's bone anatomy. The resection planes were marked under the navigation guidance, and K-wires were placed to mark the intersection and direction of the resection planes. Bone resections were then performed by a nonnavigated oscillating saw.

### 2.5. PSI-Assisted Resection

After the PSI was positioned on the predetermined bone surface at the planned bone resection site, it was pinned onto the bone surface by K-wires to prevent movement (Figure 4). The cutting platforms of the PSI guided the oscillating saw, and bone resections were achieved.

### 2.6. CAS Integrated with PSI Resection

CAS registration was performed as described before, and the PSI was placed and moved along the bone surface until the position was stable (Figure 4). Surgical navigation was then used to check the correct placement of the PSI by placing the tip of the navigation pointer on the designed checkpoints (Figure 2). The position of PSI was adjusted until it matched the planned position on the navigation display. K-wires were then placed to pin the PSI onto the bone surface (Figure 2). The bone resections were performed with the PSI guidance, and CAS was not used to determine the planned resection planes.

### 2.7. Total Bone Cutting Time

Total bone cutting time was measured for each resection. Total bone cutting time is defined as the time taken from completion of the bone exposure at the bone cutting sites to the completion of the cut. In the CAS group, this included the setup time of placement of a patient tracker, image-to-patient registration, marking the locations of the planned resection, and K-wires placement under navigation guidance. In the PSI group, this included placement of the PSI on the bone surface and the completion of the bone cut under PSI guidance. In the CAS + PSI group, this included the setup time of placement of a patient tracker, image-to-patient registration, PSI placement, intraoperative control of correct PSI placement using CAS, pinning of the PSI to the bone with K-wires, and performing the bone cuts using the PSI.

### 2.8. Postoperative Analysis

Once the osteotomies were performed, the legs, including the tumor surgical specimen obtained, were CT scanned using the same protocol as used for the preoperative CT scans. The generated 3D virtual surgical specimens were then superimposed on the 3D preoperative planning for comparison and calculation of accuracy of the achieved bone resections.

### 2.9. Location Accuracy

**Figure 5:**
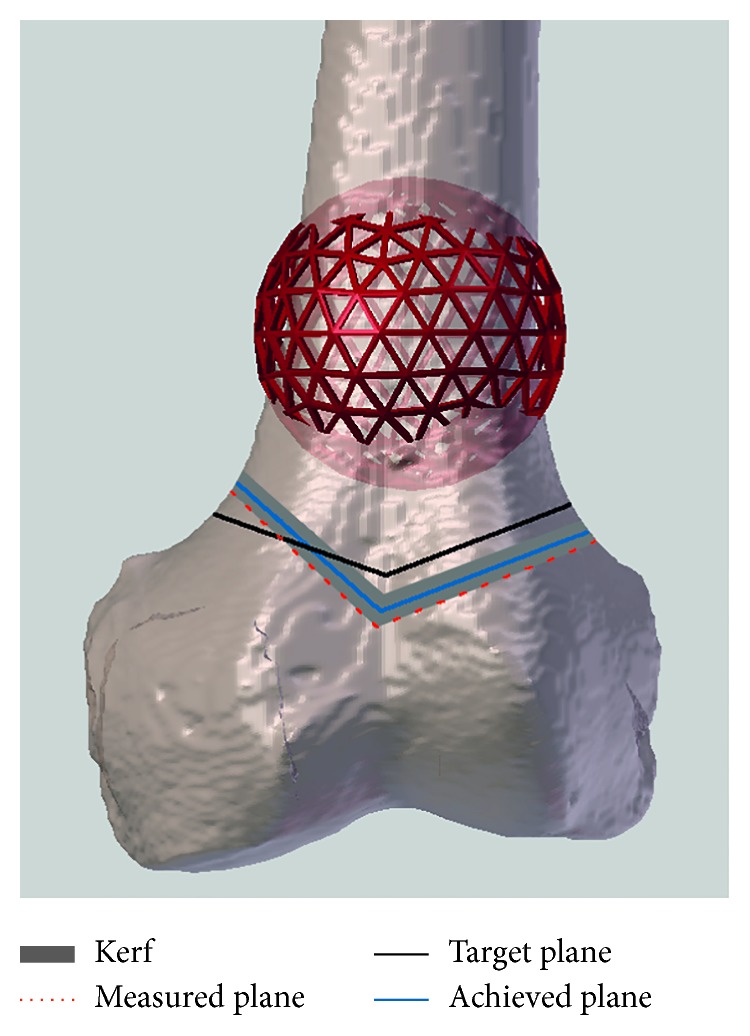
Definition of the kerf and location accuracy. The thick grey line shows the kerf, which is the bone loss that arises during an osteotomy. The saw is aligned on the target plane (black), a thin line in the middle of the kerf. The blue line defines the achieved plane of the performed resections. Location accuracy is the maximum distance (mm) between the target (black line) and achieved (blue line) planes. When the measured error or difference was on the tumor side, the error was corrected minus the kerf; when the measured error was on the healthy side, the error was corrected plus the kerf.

The location accuracy (LA) was used in this study. It is a criterion defined by the International Organization for Standardization (ISO) [25] as the maximum distance (mm) between the planned osteotomy (target) and the achieved plane (Figure 5). LA is embedding all kinds of errors, including pitch and tilt [26]. The measurements were systematically made on the healthy side (the side of the remaining bone that supports the PSI). Minimal margins were measured as well as the minimum distance between the achieved plane and the bone tumor. A kerf correction (a bone loss that arises during cutting of the bone) was performed to prevent bias. It is usually considered as 1.5 times the saw blade thickness but may vary according to teeth size and shape [27]. The saw blade used in this experiment was 0.6 mm thick. Thus, a 0.9 mm of kerf was used. The PSI did not suffer from the kerf since the measurements were made on the healthy side (the side which supported the guide) and the kerf was located on the resection side. Regarding the navigated and manual cuttings, the center of the oscillating saw was aligned on the target plane represented as a thin 2D line. The bone loss was equally spread around this line. Thus, half a kerf, 0.45 mm, has been used as a correction (Figure 5).

### 2.10. Cut-Plane Analysis

Two points were acquired at the entry line and two points at the exit line of the achieved cut. The distance between those four points and the planned trajectory was measured. The pairs of planned and achieved cuts were compared by measuring the difference in entry position and exit position. The distance between the planned and achieved cuts was calculated for both entry and exit points by calculating the perpendicular distance of the actual cut point to the planned plane.

### 2.11. Statistical Analysis

A mixed model and *t*-test were performed to evaluate the differences in location accuracy, entry and exit points of the plane, and total bone cutting time among the four groups. The data were subject to significant differences regarding mean and 95% confidence interval (95% CI). Multiple comparisons were made, and data were analyzed with and without adjustment for multiple comparisons. *p* values lower than 0.05 were considered statistically significant. Excel 2013 and SPSS version 23 were used for data management.

## 3. Results

Real-time accurate image-to-patient registration could be achieved in all navigated resections. The registration error was below 1.0 mm.

**Figure 6:**
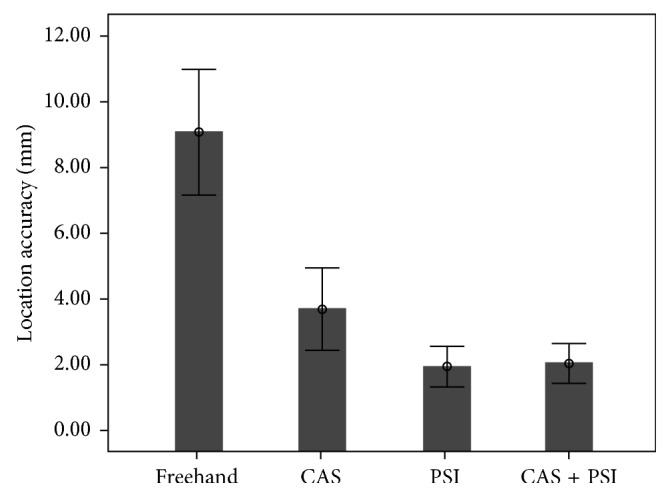
Comparison of location accuracy (mm). Mean values including the lower and upper limits of the 95% confidence interval are shown for all research groups.

The freehand group was significantly less accurate than any other groups (*p* < 0.001). No significant difference has been observed in location accuracy (LA) between the PSI and the CAS + PSI groups (*p*=0.92). The PSI and CAS + PSI groups were found to be significantly more accurate than the CAS group (*p*=0.034 and *p*=0.042, respectively) (Figure 6). The complete results of the location accuracy for each resection plane separately are available in Supplementary File 1. The mean entry and exit cut distances are significantly larger in the freehand group than any other groups (*p* < 0.05). The distance (deviation error) of the entry bone cuts was significantly smaller than that of the exit bone cuts in all of the four techniques (*p* < 0.05). When compared to the CAS group, the PSI technique showed a significantly better accuracy at the entry cut (*p*=0.003) but not at the exit cut (*p*=0.16). The CAS + PSI technique demonstrated a significantly better accuracy at the exit cut (*p*=0.03) compared to PSI but not at the entry cut (*p*=0.07).

Mean timing for each method is summarized in Table 1. The positioning of the PSI took less than one minute in all 8 procedures. Total bone cutting time of the PSI group was significantly lower than that of the CAS + PSI group (*p* < 0.001) and the CAS group (*p* < 0.001). The same significant difference has been observed between the freehand group and CAS + PSI and CAS groups (*p* < 0.001 and *p*=0.004, respectively). No difference has been found between manual and PSI groups. Finally, there was a significant difference in total bone cutting time between the CAS + PSI group and the CAS group (*p*=0.029).

## 4. Discussion

In this cadaver experimental study, we compared the surgical accuracy of different techniques in joint-preserving tumor resection around the knee joint, namely, freehand, CAS, and PSI. Furthermore, we tested a novelty: whether CAS could be used as an intraoperative quality control tool for the PSI and whether the combination of both CAS and PSI techniques could improve the surgical accuracy and thereby combine the strengths of both technologies.

The results of this cadaveric study suggested that integration of both CAS and PSI as well as PSI-assisted techniques in the simulated extremity resection could reproduce the planned resection with a mean location accuracy of less than 2 mm. PSI-assisted resections show the best location accuracy and are fastest and easiest to use. The PSI was easily positioned and found to be very stable. Thus, PSI showed better overall surgical accuracy than CAS but not in the exit bone cuts. The increased error at the exit bone cut in the PSI group may be because a PSI cannot provide visual feedback during cutting as in the CAS technique. So, adding CAS to PSI may improve the accuracy of exit cuts as shown in the results (*p*=0.03). CAS also confirmed the correct placement of PSI and therefore guided the correct orientation of the cutting platform of PSI. This can be of additional value in selected cases where soft tissue constraints hamper use of PSI. To date, there is no reliable intraoperative method to confirm the correct placement of PSI. Therefore, addition of CAS may be helpful especially in anatomical areas with insufficient landmarks or contoured bone surface where a PSI cannot be placed consistently in a predetermined position.

The results of this study are consistent with previous research of Khan et al. [28], who found a location accuracy of 2 mm while performing PSI-assisted bone tumor resections in the knee. Wong et al. showed a similar resection accuracy when using PSI in extremity bone tumor surgery [23].

To our knowledge, no previous study has been published about the integration of CAS and PSI. Integration of CAS and PSI prolonged the surgical time, but there was still a significant difference in total bone cutting time when compared to the CAS group. The greater bone cutting time for the navigation-assisted resections arises because time was needed for tracker placement, image-to-patient registration, and system calibration before the resection. Other studies [9, 29] showed a similar trend of less mean time required for the PSI-assisted bone resections. The studies showed a mean bone cutting time of around 40 min for CAS resections, which is long compared to that of the present study. The surgeons who performed the resections in this study are experienced users of CAS. Research by Farfalli et al. [29] has shown that surgical time decreased as surgeons performed more navigated procedures. This might explain the bone cutting time in the navigation group, which was lower than that reported in other studies.

This study has several limitations. First, the resections in the cadavers were based on a simple circular-shaped tumor model and an eccentric location. In other words, an ideal delineation of the tumor boundaries was used. The wide range of complex geometries such as extra-osseous components of bone tumors cannot be simulated in the real cadaver specimen. It would have been a less suitable model given the normal anatomy of the cadaver legs tested. However, the main goal of this study was to assess accuracy of bone cuts, independent of tumor size and shape. Tumor location and soft tissue mass could complicate resection planning and PSI design and placement. Second, the resections were performed in an ideal cadaveric setting with complete visualization and accessibility of the bone surface that could be stripped from all soft tissues when necessary. The bone resections might be easier when compared to a real clinical setting where soft tissue might complicate the resection; however, this only accounts for a small percentage of lesions. CAS or freehand might be an alternative here. On the contrary, if the mass is problematic for the PSI, it will be problematic for the cutting whatever the method used. Also, clinical data on pelvis have shown comparable results [7, 30]. Third, the sample size was small. A larger sample would allow a stronger conclusion, although measurements were performed using 112 bone cuts (56 entry and 56 exit cuts). Fourth, time for preoperative planning and PSI design and manufacturing was not measured. Bone cutting time decreases when using a PSI, but preoperative planning is time-consuming and could potentially delay the surgery of a tumor. However, this preparation time involves an engineer task not a surgeon task. Delays in surgery hardly happen, and time is regained easily during surgery. Fifth, except in the freehand group, the deviation errors in the groups of CAS, PSI, and CAS + PSI were less than 5 mm. Any statistically significant difference between the groups may not be clinically significant, especially when an usual 10 mm safe margin is used during the preoperative planning. However, the safe margin can be decreased to several millimeters to preserve joint, nerves, or muscle insertion. In that situation, the accuracy of the resection method is crucial to determine the safety of the planned procedure. Selection of the technique may still need to be individualized in each case, according to the surgeons' preference. Finally, there is a constant improvement going on in the CAS systems, with systems available that have navigated tools and intraoperative automatic matching. These improvements reduce setup time and aid in improving accuracy, but these complex systems are expensive and not available in every orthopedic oncology center. PSIs are fast, easy to use, and do not require training of the surgeons with complex software systems.

Clinical studies are needed to investigate whether the improved surgical accuracy can be translated into a better oncological outcome and to investigate how the depth of the cuts, the width of the cutting platform of the PSI, or adding a cutting slit with metal sleeve influence the surgical accuracy.

## 5. Conclusion

In this experimental cadaveric study, we showed that PSI-assisted resections have the best overall surgical accuracy and are fast and easy to use. CAS could be used as an intraoperative quality control tool for the PSI, and integration of CAS and PSI is possible. Adding CAS to PSI did however not improve overall surgical accuracy, but CAS might improve the accuracy of exit cuts. Although the results were significantly different, both CAS and PSI seem to be complementary in improving surgical accuracy and are not mutually exclusive. Surgeons should choose the technique that is most suitable for their patient, based on the patient and tumor specifics. Further research is needed to investigate the clinical efficacy of integrating CAS and PSI and evaluate the accuracy of PSI and CAS alone.

## Figures and Tables

**Table 1:** Location accuracy (56 entry and 56 exit cuts) and mean bone cutting time for each technique.

Parameters		Freehand	CAS	PSI	CAS + PSI
Mean location accuracy (mm) (95% CI)		9.2 ± 3.3 (8.0; 10.3)	3.6 ± 2.1 (2.5; 4.8)	1.9 ± 1.1 (0.8; 3.0)	2.0 ± 1.0 (0.9; 3.1)

Plane direction	Entry cuts (mm)	4.3 ± 2.7	2.1 ± 1.3	1.2 ± 0.9	0.8 ± 0.5
	Exit cuts (mm)	6.8 ± 3.4	3.1 ± 2.2	2.3 ± 1.5	1.6 ± 0.8

Mean bone resection time (minutes)		6.8 ± 1.1	17.0 ± 2.4	4.8 ± 1.0	12.8 ± 3.8

Standard deviation is listed for each value. 95% CI is given for the LA.

## Data Availability

The data on location accuracy for each resection and time measurements of each procedure to support the findings in this study are included within the supplementary file.

## Supplementary Materials

Supplementary Materials The data on location accuracy for each resection and time measurements of each procedure.
