# Supplementary material for: A Cadaveric Comparative Study on the Surgical Accuracy of Freehand, Computer Navigation, and Patient-Specific Instruments in Joint-Preserving Bone Tumor Resections
Source: Sarcoma. 2018 Nov 13;2018:4065846. doi: 10.1155/2018/4065846 (PMC6260549; doi:10.1155/2018/4065846)
Supplement: Supplementary Materials — The data on location accuracy for each resection and time measurements of each procedure. [file 4065846.f1.pdf]

Supplementary file

Location accuracy (mm)			Freehand	Freehand corrected	CAS	CAS corrected	PSI	CAS+PSI
Left	Tibia	Distal plane	8.65	9.1	2.1	2.55	0.74	0.85
		Proximal lateral	15.13	15.58	2.36	1.91	1.1	2.33
		Proximal medial	6.55	7	3.45	3.9	0.55	0.76
		Proximal central	11.21	11.66	0.99	1.44	0.93	1.94
	Femur	Proximal plane	9.3	10.75	7.4	7.85	2.61	1.66
		Distal lateral	10.26	11.71	7.54	7.09	2.58	1.55
		Distal medial	13.16	11.71	2.39	1.94	1.38	1.16
Right	Tibia	Distal plane	4.95	5.4	3.86	3.41	3.2	2.75
		Proximal lateral	7.8	8.35	5.69	6.14	1.13	1.6
		Proximal medial	3.28	3.83	1.46	1.91	2.17	2
		Proximal central	9.92	8.47	1.79	2.24	4.4	4.61
	Femur	Proximal plane	11.85	12.4	3.32	3.77	2.04	1.12
		Distal lateral	5.18	4.63	4.46	4.91	1.97	2.25
		Distal medial	8.15	7.6	2.41	1.96	1.92	3.26
		Mean (mm)		9.16		3.64	1.91	1.99
		Standard deviation		3.34		2.10	1.06	1.04
		95% Confidence interval (CI)		8.02-10.29		2.52-4.77	0.78-3.04	0.86-3.11

Table 1 - Location accuracy in mm defined as a maximum deviation from the target or planned plane. The location accuracy in the freehand and navigation-assisted resections are corrected by half a kerf, 0.45 mm, minus or plus according to the direction of the measured error (towards the resection part or not). Standard deviation (sd) and 95% confidence interval (CI) are shown for the means of all groups.
